# The relationship between Anti-Müllerian hormone and other reproductive parameters in normal women and in women with polycystic ovary syndrome

**Published:** 2013-06-25

**Authors:** N Koutlaki, M Dimitraki, S Zervoudis, C Poiana, A Psillaki, I Nikas, A Liberis, C Badiu, V Liberis

**Affiliations:** * Department of Obstetrics and Gynecology, University Hospital of Alexandroupolis, Democritus University of Thrace, Alexandroupolis, Greece; ** Rea Maternity Hospital and TEI Tech. University, Athens, Greece; *** Carol Davila University of Medicine, Bucharest, Romania

**Keywords:** Anti-Müllerian hormone, menarche, ovarian volume, testosterone, PCOS

## Abstract

Objective: To correlate Anti-Müllerian hormone (AMH) levels with years since menarche as well as to investigate the AMH relationship with ovarian morphology and levels of androgens in healthy normo- ovulatory women and in women with polycystic ovary syndrome (PCOS).

Design: Prospective clinical study.

Setting: University Hospital of Alexandroupolis, Lito Maternity Hospital.

Patients: Forty two healthy normo-ovulatory women and sixty one women with PCOS, recruited on the basis of the classic PCOS criteria (Rotterdam consensus meeting definition of PCOS (ESHRE/ASRM, 2004).

Interventions: Fasting blood was obtained from all subjects in the early follicular phase (days 5-6) after spontaneous or induced menses (in PCOS), and transvaginal ultrasound examination was performed. Main outcome measures: Assessment of values for follicular stimulating hormone (FSH), testosterone (T), AMH, as well as assessments of years since menarche and ovarian volume.

Results: AMH had a statistically significant positive correlation with the ovarian volume (r =0,623, r =0,579 P<0.01) and negative correlation with years since menarche (r =-0,766, r =-0,796 (P<0.01). In women with PCOS, AMH and years since menarche had a significant correlation with testosterone (r =0,477, r = -0,527, P<0.01)

Conclusions: This study underlines the relation between AMH and years since menarche as well as the AMH differences in relation with certain clinical or endocrine characteristics between normal and PCOS women.

## Introduction

Anti-Müllerian hormone also known as mullerian inhibiting substance (MIS), is a member of the large transforming growth factor-p (TGFP) multigene family of glycoproteins, that are involved in the regulation of growth and differentiation. AMH is produced by fetal Sertoli cells at the time of testicular differentiation, and induces regression of the Müllerian ducts [**1**]. In the absence of AMH, the Müllerian ducts develop into the uterus, fallopian tubes and the upper part of the vagina [**2**]. In the ovary, AMH is produced by the granulosa cells of early developing follicles and seems to be able to inhibit the initiation of primordial and FSH-induced follicle growth. Interestingly, AMH is expressed in human follicles from the primary follicular stage towards the antral stage, immediately after recruitment right up to the selection stage (4-6 mm diameter) [**3**]. Therefore, serum levels of AMH are proportional to the number of developing antral follicles in the ovaries and may represent both the quantity and quality of the ovarian follicle pool [**4**]. A number of studies have shown serum AMH levels to be increased in women with polycystic ovary syndrome (PCOS) [**5,6**]. PCOS ovaries contain 2-3-fold the normal number of follicles, from the time when they start growing to a size of 2-5 mm (antral follicles) and PCOS follicles stop growing and developing when they reach 4-7 mm in diameter [**7-9**]. Thus, elevated serum AMH levels in women with PCOS are the result of increased synthesis by developing antral follicles in the polycystic ovaries. Moreover, AMH’s role in follicular growth implies its potential value as a marker for the type and extent of ovarian dysfunction in anovulatory women with polycystic ovary syndrome (PCOS). There is a debate on the association of androgen and AMH serum levels in women with polycystic ovary syndrome (PCOS). It has been demonstrated that androgens promote both theca interna cells and granulose cells proliferation [**10,11**]. This effect on folliculogenesis predominates in small follicles, probably, due to their richness in androgen receptors as it has been reported that the androgen receptor gene expression is the highest in GC of preantral and antral follicles [**12**]. Additionally, the balance of studies shows that AMH, could be implicated in the hyperandrogenism of PCOS as the detection of AMH type II receptor (AMHRII) in theca interna cells of maturing follicles [**13**] by in situ hybridization, that could lend support to a paracrine effect of AMH on these cells. Because AMH constitutes a marker for the number of small follicles, its correlation with ovarian volume and the number of follicles present in the ovary is not surprising. Indeed, serum AMH levels have an excellent correlation with the number of antral follicles (antral follicular count -AFC) [**14,16**]. Some authors [**17**] have also associated AFC and ovarian volume (OV) and have further negatively correlated AFC and OV with age, indicating that ovarian follicles decreased with increase in age. This prospective study was designed to investigate the relationship between Anti-Müllerian hormone (AMH) levels and ovarian volume (OV) and to determine whether these markers of ovarian reserve correlate with serum androgen levels and years since menarche (gynecological age) in women with PCOS versus normo-ovulatory women.

## Materials and Methods

### Subjects

Forty-two healthy normo-ovulatory women and a total of 62 women with PCOS were recruited between January 2009 and May 2010 in the Ob/Gyn. Department of the University Hospital of Alexandroupolis and Lito Maternity Hospital. All subjects were of 25-35 years of chronological age. The Institutional Review Board of both Institutions approved the study. For the enrollment of the PCOS subjects, the Rotterdam criteria (2004) [**18**] were used and the diagnosis of PCOS was based on at least two of the following three criteria: oligomenorrhea (>8 spontaneous menstrual cycles per year for at least 3 years before enrollment) or amenorrhea, biochemical hyperandrogenemia (serum total testosterone level >0.8 ng/ml), and polycystic ovaries (>12 follicles in the 2–9 mm range and/or an ovarian volume >10 ml per ovary by vaginal ultrasound). Hyperprolactinaemia, thyroid dysfunction, Cushing’s syndrome, congenital adrenal hyperplasia and other disorders (current or previous pregnancy (within 1 year of enrollment), adrenal tumor, ovarian tumor, autoimmune disease, malignancy, central nervous system disease, current or previous use of oral contraceptives (within 6 months of enrollment), or the use of medications (known to affect the hypothalamic–pituitary–ovarian axis) were excluded before enrollment into the study. 

### Clinical protocol

 Overnight fasting blood samples were collected randomly from all subjects in the early follicular phase (days 5-6) after spontaneous or induced menses (in PCOS), and transvaginal ultrasounds were performed. 

 The hormonal study included a baseline plasma determination of AMH, LH, FSH, E2, T. 

Years since menarche were reported for each subject. 

 The presence of hirsutism was determined by applying the Ferriman–Gallway score criteria [**19**]. A score value >8 was considered positive for the presence of hirsutism. 

 Transvaginal US (TVUS) was performed on each patient by using a 6.5 MHz endovaginal probe. The ultrasound measurements were obtained in real-time by a single physician according to a standardized protocol. Ovarian volume was calculated for each ovary by using the formula for a prolate ellipsoid: P/6 X (D1X D2 X D3), where D represented the maximum diameter in transverse, antero-posterior and long section (or 0.5 X length X width X thickness) [**20**]. Ovarian volume per ovary (OV) was defined as the average of the ovarian volume obtained from both ovaries. 

### Assay methods

Blood samples were centrifuged immediately after collection and then stored at -20oC until time of assay. All hormone concentrations were determined by using the electrochemiluminescence immunoassay “ECLIA" (for use on Elecsys and Cobas and immunoassay analyzers). Serum AMH levels were assessed by using a competitive inhibition enzyme immunoassay technique for the in vitro quantitative measurement of AMH (ABIN414951).

### Statistical analysis

The data are presented as the median with 5–95th percentiles. The Shapiro–Wilk test was used to identify whether all the variables were normally distributed. Pearson correlation coefficients were calculated to determine the correlations between the variables. Multiple linear regression analysis was performed by using AMH as the dependent variable, and FSH, ovarian volume, years since menarche, age and testosterone, as the independent variables. A p-value of 0.05 was considered statistically significant. Data were stored and analyzed with the use of Statistical Program for Social Sciences release 15.0 (SPSS Inc., Chicago, IL, USA). 

## Results

The clinical and hormonal characteristics of the subjects are depicted in Table 1. 

**Table 1:** Clinical and hormonal characteristics of the group of normal women and the group of women with PCOS

	Normal (n=42)		PCOS (n=61)	
	Median	Range(5-95%)	Median	Range(5-95%)
Age (years)	29	25-35	29,5	25-35
FSH (mIU/ml)	5,785	3,65-8,89	5,825	3,64-8,56
LH (mIU/ml)	5,085	2,45-8,67	12,045	3,79-18,35
E2 (pg/ml)	24,5	16-82	32	19-23
T (ng/ml)	0,31	0,1-0,77	0,825	0,42-1,87
Menarche (years)	16,2	11,5-23,2	15,75	10,3-22,1
OV (ml)	8,435	5,02-13,89	9,88	5,36-16,46
AMH (pM/L)	33,09	11,15-68,75	45,12	10,67-99,78
Menarche, years since menarche; OV, ovarian volume per ovary; AMH, anti-Mullerian hormone

 AMH showed a significant correlation with OV, testosterone and years since menarche in both groups of women (P<0.01) (**Table 2,3**). 

**Table 2:** Correlations among variables in normal women

	AMH	T	OV	MENARCHE	FSH
AMH	-				
MENARCHE	-0,766 (P<0,01)	-0,201 NS	-0,827 (P<0,01)	-	
OV	0,623 (P<0,01)	0,417 (P<0,01)	-		
T	0,085 NS	-			
FSH	-0,443 (P<0,01)	-0,025 NS	-0,589 (P<0,01)	0,643 (P<0,01)	-
AGE	-0,802 (P<0,01)	-0,130 NS	-0,804 (P<0,01)	0,960 (P<0,01)	0,661 (P<0,01)
Values are expressed as the Pearson correlation coefficient (r); P-values are indicated in parentheses; NS indicates non-significance at P < 0,01

**Table 3:** Correlations among variables in women with PCOS

	AMH	T	OV	MENARCHE	FSH
AMH	-				
MENARCHE	-0,796 (P<0,01)	-0,527 (P<0,01)	-0,780 (P<0,01)	-	
OV	0,579 (P<0,01)	0,788 (P<0,01)	-		
T	0,477 (P<0,01)	-			
FSH	-0,515 (P<0,01)	-0,355 (P<0,01)	-0,6,18 (P<0,01)	0,692 (P<0,01)	-
AGE	0,798 (P<0,01)	-0,525 (P<0,01)	-0,754 (P<0,01)	0,970 (P<0,01)	0,676 (P<0,01)
Values are expressed as the Pearson correlation coefficient (r); P-values are indicated in parentheses; Correlation is significant at the 0,01 level. estimate

AMH had a significant negative association with both FSH and age (P<0.01). In PCOS group AMH and years since menarche had a significant correlation with testosterone (P<0.01). Years since menarche had a significant negative association with ovarian volume in both groups. 

Multivariable linear regression analysis was used to determine the significant independent association between AMH and FSH, age, years since menarche, testosterone or ovarian volume. In the group of normal women only age was more related to AMH, without reaching statistical significance (b -0,924; P=0.17) in the final model after the introduction of age, FSH, OV, menarche and total T as independent variables (**Table 4**). 

**Table 4:** Multiple linear regression models of dependent variable AMH for normal women and women with PCOS

	Normal		PCOS			
	Model R2=0,659		Model R2=0,673			
	B	Beta	P	B	Beta	P
FSH	1,615	0,157	0,246	0,3	0,016	0,884
AGE	-4,233	-0,924	0,17	-3,123	-0,359	0,267
OV	-0,103	-0,015	0,941	-3,769	-0,344	0,065
T	-2,458	-0,025	0,835	18,980	0,252	0,062
MENARCHE	0,012	0,003	0,995	-4,950	-0,595	0,088
Covariates considered for selection in each model: FSH, age, ovarian volume, testosterone and years since menarche.

 However, using multiple linear regression analysis, in the PCOS group, OV (b 0.344; P =0.065), T (b 0.252; P=0.062) and menarche (b -0.59; P=0.088) were related to AMH. There was a weaker relation between other variables and AMH.

## Discussion

Since the number of ovarian follicles decreases with age, AMH is a good indirect marker of the quantitative aspects of the ovarian reserve reflecting all follicles that have made the transition from the primordial follicle pool to the growing pool, being thus, a marker for ovarian ageing. 

 Our results clearly showed a strong negative correlation between the years since menarche and AMH, both in women with PCOS and in normo-ovulatory women, indicating that the ovarian follicle pool decreases with the increase in the gynecological age.

 Several studies have demonstrated an association between AMH and age. While examining cross sectional changes in early follicular (days 3-5) AMH levels among 238 infertile women, Tremellen et al. [**21**] found that AMH levels remained relatively stable from 18 to 29 years of age, followed by a 50% decline in AMH levels between 29-37 years of age (decline to 1 ng/mL by age 37). Consistent with the above study, De Vet et al. [**22**] prospectively followed 41 normo-ovulatory women of reproductive age (mean age 29 years), over a mean of 2.6 years and noted a 38% decline in mean serum day 3 AMH levels (from 2.1 ng/mL to 1.3 ng/mL). Furthermore, Van Rooij et al. [**23**] noted the mean day 3 serum AMH to decline to 58% (1.2 ng/mL to 0.5 ng/mL) in a prospective longitudinal study of 81 healthy fertile women of reproductive age (mean age 39.6 years) over an interval of 4 years. 

 The findings of the present study suggest that the years since menarche correlate negatively with serum AMH levels although the strongest correlations have been found in women with PCOS. Other study groups have shown a direct correlation between the gynecological age and AMH serum levels. Three years ago, Nardo et al. [**24**] demonstrated that AMH and AFC correlated negatively with age (r = −0.30, p < 0.001 and r = −0.27, p = 0.001 respectively) and number of years since menarche (r = −0.23, p = 0.007 and r = −0.21, p = 0.015 respectively).

 On the other hand, our data indicate negative correlation between OV and years since menarche in both study groups. Numerous studies [**25**] have shown that the most important factor for the ovarian size was age. Wallace and Kelsey [**26**] demonstrated that the ovarian volume in women aged 25-51 years accurately reflects the number of primordial follicles remaining and reproductive age. Pavlik et al. [**27**] calculated the mean ovarian volume according to the age for each decade of life and found a statistically significant decrease in the ovarian volume with each decade of life from age 30 to age 70. However, Erdem et al. [**28**] demonstrated that age was weakly correlated with OV (r = - 29, P < 0.05). In contrast to the above observations, in another study [**29**], the authors evaluating the ovarian size in 1888 infertile women undergoing IVF, found that the total ovarian volume did not change significantly across age groups (23-45 years). However, the results of this study manifested significantly decreasing ovarian size by age, especially between age 25-30 year (youngest group) and 41-45 years (oldest group). It has been hypothesized that the increased number of small follicles is due to the trophic effects of androgens, whether increased locally in the ovary as in PCOS, or systematically.

 In this new series of patients, we provided confirmation that US findings (OV) are positively related to androgen levels and AMH serum levels in PCOS group (and years since menarche). It has been reported [**30**] that the 2-5 mm follicle number at ultrasound was positively correlated with the serum testosterone and androstenedione levels in patients with PCOS. Puzigaka et al. [**31**] demonstrated a good correlation between OV and androgen levels. Interestingly, Turhan et al. [**32**] suggested the importance of ovarian volume measurement as an indicator of androgen production in PCO women, reporting a significant positive correlation between ovarian volume and serum LH, testosterone, androstenedione, DHEAS levels. In contrast to these findings, Nardo et al. [**33**] failed to find any correlation between T levels and US parameters in 23 PCOS subjects studied by 3D TVUS.

 In conclusion, we demonstrated that gynecological age (years since menarche) had a better negative correlation with AMH and OV in women with PCOS than in normo-ovulatory women. In the same group of women, AMH and OV correlated significantly with androgen serum levels and gynecological age.
